# Electroencephalographic frequency activity of patients with bipolar disorder

**DOI:** 10.1192/j.eurpsy.2023.1475

**Published:** 2023-07-19

**Authors:** M. Mnif, L. Triki, N. Smaoui, D. Jardak, S. Omri, I. Gassara, R. Feki, M. Maalej, J. Ben Thabeut, L. Zouari, N. Charfi, K. Masmoudi, M. Maalej

**Affiliations:** 1 Psychiatry C department, Hedi Chakeur Hospital; 2 Functional exploration department, Hbib Borguiba Hospital, Sfax, Tunisia

## Abstract

**Introduction:**

Bipolar disorder (BD) is a common psychiatric condition. However, it is underdiagnosed. Electroencephalography (EEG) has been proposed as a neurophysiological biomarker to delineate psychotic disorders.

**Objectives:**

To compare the EEG tracings of patients with BD with those of normal subjects to aid in diagnosis.

**Methods:**

This was a cross-sectional, descriptive, and analytical case-control study conducted with patients followed for BD in the psychiatry "C" department at the Hedi Chaker hospital in Sfax. Patients were assessed by the Young Mania Scale (YMRS), the Hamilton HDRS-17 Scale, and the Medication Adherence Report Scale (MARS). Healthy controls were included. All participants benefited from an EEG. It was undertaken in resting eyes closed testing conditions at the service of the functional exploration at the Habib Bourguiba hospital in Sfax. The powers of each band were measured using the power spectral density method called absolute power (AP). Statistical analyses were carried out.

**Results:**

Fifteen bipolar patients and 15 healthy controls, all male, were included. The average age of bipolar was 36.07 ± 10.50 years. The one of health control was 47.93 ± 15.61 years. There were no significant differences in age between bipolar patients and healthy controls. The mean scores on the HDRS-17 and YMRS and MARS scales were 2.73±2.08, 1.67±3.53 and 5.8±2.83 respectively.

At the quantitative EEG, differences appeared to be insignificant. There was an overall decrease in AP for alpha band particularly in the parietal and occipital lobes in bipolar patients (158,84 ± 447,71 μV2 and 188,21 ± 415,55 μV2 respectively) compared to controls (335,15 ± 994,73 μV2 and 400,24 ± 1109,95 μV2 respectively). An overall increase in AP for delta and beta was found for bipolar patients compared to controls.

**Conclusions:**

Our main finding was a higher delta and beta frequency activity, and lower alpha frequency activity in bipolar patients compared to controls, which may aid in the diagnosis of this disorder.

**Disclosure of Interest:**

None Declared

